# Melanotic medullary carcinoma of thyroid – report of a rare case with brief review of literature

**DOI:** 10.1186/1746-1596-3-2

**Published:** 2008-01-11

**Authors:** Kamaljeet Singh, Mehar C Sharma, Deepali Jain, Rajinder Kumar

**Affiliations:** 1 Department of Pathology, All India Institute of Medical Sciences, New Delhi, India; 2 Department of Surgery, All India Institute of Medical Sciences, New Delhi, India

## Abstract

**Background:**

Melanin production in medullary carcinoma is extremely uncommon.

**Case presentation:**

We report a rare variant of medullary carcinoma of thyroid with melanin production in a 52-year-old woman who presented with swelling in the thyroid of 3 months duration. This tumor recurred thrice in two years after surgery and patient died with metastasis. Microscopic examination showed typical morphology of medullary carcinoma with numerous cells loaded with melanin pigment as confirmed by bleached Fontana-Masson, negative iron and immunohistochemical stains. Tumor cells were diffusely immunopositive for calcitonin, HMB-45, chromogranin, synaptophysin, CEA but showed focal paranuclear dot positivity for cytokeratin. No C-cell hyperplasia was seen in the adjacent thyroid gland. Nature of the pigment was further confirmed on ultra structural examination.

**Conclusion:**

Melanotic medullary carcinoma is an extremely uncommon entity. There is a need to report more number of cases in the literature for exact categorization and prognostication of this subtype of medullary carcinoma.

## Background

Hazard et al segregated medullary carcinoma of thyroid (MTC) as a distinct entity in 1959 [1]. Calcitonin production by tumor cells is distinct and proves its origin from C-cells [2]. Medullary carcinoma cells have a confirmed ability to have a multi directional differentiation. Tumor cells are known to produce array of hormonal and non-hormonal products including mucin, peptides and amines [2]. Melanin production in medullary carcinoma is extremely uncommon and to the best of our knowledge only nine such cases have been reported in the literature [3-11] (Table 1). Melanocytic differentiation with melanin production is very rare, thus needs to be documented in order to know the exact behavior of this variant.

**Table 1:** Summary of published cases of melanotic medullary carcinoma thyroid

**S. No.**	**Author & Year**	**Age & Sex**	**Duration of symptoms**	**Histology**		**Pigmentation**	**Follow up**
1.	Marcus JN 1982 [3]	53/M	15 days	Classic	Dendritic cells	Dendritic cells	No evidence of recurrence or metastasis 2.5 years after surgery
2.	Posen JA 1984 [4]	38/F	5 years	Classic	--	Diffuse	Not mentioned
3.	Eng HL 1989 [5]	68/F	1 month	Classic	Dendritic cells	Dendritic cells	Not mentioned
4.	Kimura N 1989 [6]	62/M	2 years	Classic	Glandular	Both components	2 years metastasis
5.	Beerman H 1990 [7]	48/F	15 days	Classic	Spindle cells	Spindle cells	13 months died with metastasis
		36/F	14 years	Classic	--	Diffuse	20 months
6.	Romdhane KP 1995 [8]	51/M	10 years	Paraganglioma like	Dendritic cells	Dendritic cells	No evidence of recurrence or metastasis 1 year after surgery
7.	Ikeda T 1998 [9]	72/F	2 months	Paraganglioma like	Sustentacular cells	Sustentacular cells	Not mentioned
8.	Singh ZN 1999 [10]	55/F	4 months	Typical	--	Diffuse	Presented with LN metastasis; No evidence of metastasis after 1 year
9.	De Lume MA 2001 [11]	20/M	9 months	Classic	--	Diffuse	Not mentioned
10.	Present case	52/F	3 months	Classic	--	Diffuse	24 months, recurred thrice, metastasis to skull bones and died

## Case presentation

A 52-year-old female presented with swelling in the neck, in the thyroid region, of 3 months duration. She complained of difficulty in swallowing and pain in the swelling for the last fifteen days. Clinically, she was euthyroid and there was no past history of exposure to radiation. Routine blood examination and serum chemistry revealed no abnormalities. Serum T3, T4 and TSH levels were normal. Renal function and liver function tests were normal.

There was no family history of thyroid disease. She was a known hypertensive on treatment. On examination a 3 × 2 cm lump was palpable in the lower part of neck, more so on the right side. This swelling moved with deglutition. No lymph nodes were palpable in the neck. No clinical evidence of abnormal parathyroid activity, Cushing syndrome, subcutaneous nodules or mucosal neuromas was present.

**Figure 1:**
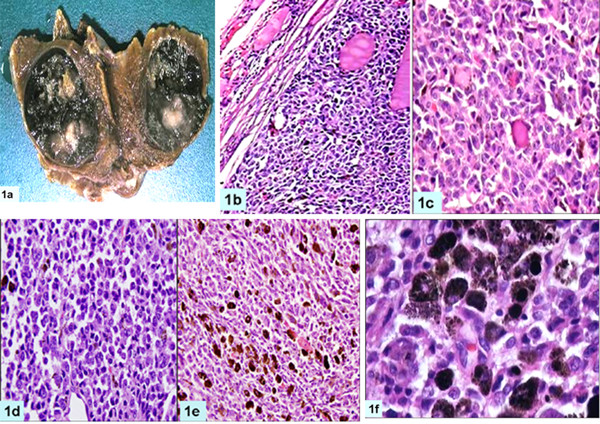
Gross photograph showing cut surface of right lobe of thyroid demonstrating a well circumscribed black colored nodule, (1a); photomicrograph shows encapsulated nodule compressing adjacent normal thyroid (1b, H&E, ×100); caught up normal thyroid follicles in the tumor (1c, H&E, ×200); higher magnification demonstrating plasmacytoid appearance of tumor cells (1d, H&E, ×200); tumor cells containing brownish black pigment and focal areas of spindling (1e, H&E, ×200); high magnification shows tumor cells laden with melanin (1f, H&E, ×400)

Computerized tomography (CT) scan neck showed a 3.5 × 2.5 cm hyper dense lesion in the right lobe of the thyroid without any associated lymphadenopathy. Fine needle aspiration cytology (FNAC) was suggestive of a poorly differentiated carcinoma. On laryngoscopic examination, bilateral vocal cords were mobile. Near total thyroidectomy was done without lymph node dissection. Post-operative period was uneventful. Unfortunately, serum calcitonin level was not estimated preoperatively but postoperative serum calcium and calcitonin levels were within normal limits. Thyroid weighed 24 gm and thyroid capsule was intact. Cut surface showed a single nodule measuring 3.1 × 2.2 × 1.1 cm within the right lobe of thyroid. Nodule was partially encapsulated but well demarcated from adjacent normal appearing thyroid. Cut section of nodule was soft homogeneously black in color with grey white foci (Fig 1a). No satellite nodule was seen in the rest of the thyroid. Lymph nodes were not found in the specimen.

Tissue was fixed in 10% neutral buffered formalin, routinely processed and paraffin embedded. Five micron thick sections were cut for hematoxylin and eosin staining and immunohistochemistry. Immunohistochemical staining was done by streptavidin-biotin peroxidase complex method and a panel of antibodies was used to characterize the tumor. These include thyroglobulin (dil 1:50), thyroid transcription factor (TTF, dil 1:100), synaptophysin (dil 1:50), carcinoembryonic antigen(CEA, dil 1:100), melan A(dil 1: 100), epithelial membrane antigen (EMA, dil 1:50), pancytokeratin (CK; dil 1:100), cytokeratin 19 (dil 1:100), S-100 protein (dil 1:100), vimentin (dil 1:100), chromogranin (dil 1:50), calcitonin (dil 1:50), HMB 45 (dil:100), p53 (dil 1:50), bcl-2 (dil 1:50) and MIB-1 (dil 1:50). All antibodies were obtained from M/s Dako Patts, Denmark except p53 and TTF which were procured from M/s Novocastra UK and M/s Neomarker, USA respectively. High temperature antigen retrieval using microwave was carried out by immersing the sections in 10 mM citrate buffer (pH 6.0) and heating inside a 750 watt microwave oven in full power for 30 minutes except for chromogranin, HMB-45, S-100 and EMA.

**Figure 2:**
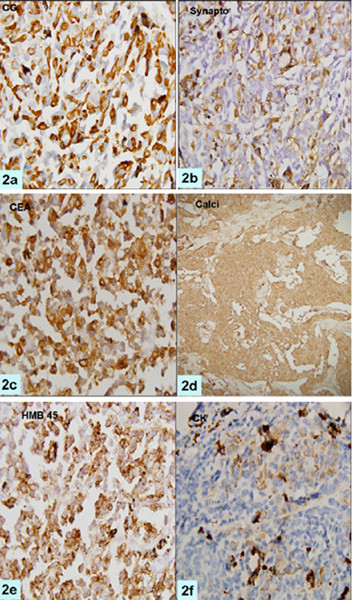
Photomicrographs showing tumor cells to be immunopositive for chromogranin A, synaptophysin, CEA, Calcitonin, HMB-45, and pan-CK (a-f, ×200 each).

**Figure 3:**
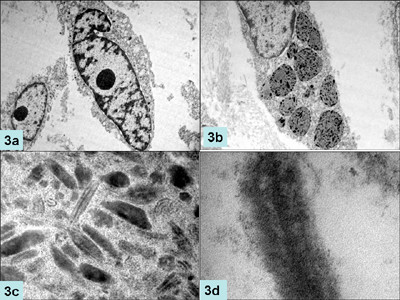
Electronmicrographs showing oval to spindle shaped cells with prominent nucleoli (a, ×2250 original magnification) packed with compound melanosomes and premelanosomes (b, ×6250); higher magnification of premelanosomes demonstrating internal structure (c, ×8250) and electron dense neurosecretory granules (d, ×6250).

MIB-LI was calculated as percentage positive nuclei in the highest labeled areas. For electron microscopy, the tissue was fixed in 2.5% glutaraldehyde, post-fixed with 1% osmic acid and embedded in epoxy resin. Routine thick sections were cut to select representative area for ultra-thin sectioning. Ultra-thin sections were double stained with uranyl acetate and lead citrate and examined under a transmission electron microscope (TEM, Morgagni 268, Holland). Microscopic examination showed a cellular tumor arranged in solid sheets, nests and trabecular pattern with few residual normal thyroid follicles (Fig 1b–f). Tumor cells were polygonal, round to oval, at places showing spindling. Cellular outlines were indistinct with moderate to abundant amphophilic cytoplasm. Nuclei were vesicular and showed moderate pleomorphism, multi-nucleation and conspicuous nucleoli. Frequent mitoses were present but necrosis was not seen. Scant perivascular eosinophilic amorphous material was present which was reactive to Congo red stain and showed apple green birefringence on polarization. Brown-black, coarse intracytoplasmic pigment was present in the tumor cells (Fig. 1e–f) which bleached with Masson-Fontana stain and it was negative for iron stain. Melanin was present in all types of tumor cells. Tumor cells showed diffuse immunopositivity for calcitonin, HMB-45, carcinoembryonic antigen, synaptophysin, chromogranin, vimentin and epithelial membrane antigen but focal paranuclear dot-like positivity for cytokeratin (Fig 2a–f), and were negative for thyroglobulin, S-100, melan-A and TTF (Table 2). Electron microscopy revealed abundant compound melanosomes, premelanosomes in various stages of melanogenesis and sparse membrane bound dense core neurosecretory granules measuring 50–200 nm along with intermediate filaments (Fig 3). Patient was discharged on fourth post operative day and was doing well.

**Table 2:** Summary and comparison of immunohisochemical profile of the previously reported cases

IHC	Present case	*3	*4	*5	*6	*7	*8	*9	*10	*11
Calcitonin	+	+	+	+	+	+	+	+	+	+
HMB-45/Melanin stain	+	+	+	+	+	+	+	-	+	+
Carcinoembryonic antigen	+				+			+		
Synaptophysin	+									
Chromogranin	+				+		+	+		+
Epithelial membrane antigen	+									
Vimentin	+									
Cytokeratin	+							+	+	
S-100	-				-		+	+	+	
Melan-A	-									

*REFERENCE NUMBER

+ – positive,

- – negative

Follow up: However in the follow up, she developed local recurrence twice after 10 months and 18 months of operation in the soft tissue of neck. Serum calcitonin levels were within normal range. Excision of recurrent nodules showed medullary carcinoma without pigment production. After 3 months of second recurrence, she developed multiple nodules at the same site and surgical excision was done in another hospital. After this surgery she had multiple metastases to the scalp and died after 24 months of first surgery. Mutational analysis for ret oncogene could not be done.

## Discussion

Medullary carcinoma of thyroid (MTC) is a tumor of thyroid parafollicular C cells, which are derived from neural crest. MTC can mimic any tumor structurally and functionally and has also been described as solid carcinoma, hyaline carcinoma and C-cell carcinoma [1]. Classically tumor cells are round to polygonal with amphophilic granular cytoplasm and have medium sized nucleus. Tumor cells are spread in solid sheets separated by highly vascular stroma, hyalinised collagen and amyloid [11]. Unusual varieties of MTC described are melanin producing MTC [3-11], true papillary form [12], mucinous or amphicrine form [13], clear cell variant [14], small cell variant [15] and giant cell type [16].

Our patient was a 52-year-old lady with mass in the thyroid region in the neck for three months. Histopathologic examination showed features of medullary carcinoma with melanin production. Mitoses were frequent (>10/hpf) in some foci which is unusual for MTC. Multinucleated tumor giant cells were present. These giant cells had hyper chromatic, pleomorphic nuclei with prominent amphophilic to eosinophilic nucleoli. Positivity for Fontana-Masson and negativity for iron stain confirmed pigment to be melanin. Tumor cells were positive for calcitonin which is the most specific marker for parafollicular cells [1] and proved beyond doubt that this was a melanin producing MTC and not a metastatic melanoma. Positive immunostaining for cytokeratin, chromogranin A, synaptophysin and carcinoembyonic antigen further buttressed above diagnosis. MTCs are generally negative for thyroglobulin as has been seen in the present case [17]. However mixed medullary carcinomas are known for thyroglobulin immunopositivity secondary to follicular differentiation. It has been hypothesized that mixed medullary carcinoma of the thyroid may be derived from common stem cells capable of differentiating into both follicular and parafollicular tumor cells [18]. Conversely, Volante et al [19] raised the possibility that trophic factors secreted by neoplastic and host cells might have a significant role in the tumorigenesis of mixed MTC. The demonstration of immunoreactive calcitonin within the neoplastic cells and the lack of thyroglobulin reactivity further support the diagnosis of a pure medullary carcinoma. Ultra structurally, tumor cells contained abundant melanosomes and premelanosomes but sparse neurosecretory dense core granules.

Black thyroid glands have been described in patients of cystic fibrosis, obstructive lung diseases and in patients who received minocycline for long duration. Pigmentation in these conditions is secondary to accumulation of lipofuscin. Presence of melanin in this case along with absence of clinical features ruled out these conditions. Metastatic melanoma has to be excluded before considering melanin producing medullary carcinoma as the primary diagnosis. Melanoma is the most common metastatic tumor in the thyroid [20,21]. Routine hematoxylin and eosin stain may not help much in differentiating melanoma from MTC with melanin production. In the present case the tumor cells showed pleomorphism with prominent nucleoli, foci of high mitotic counts and the melanin pigment. Features favoring MTC were organoid pattern; moderate to abundant eosinophilic cytoplasm and scant amyloid like material, however melanoma can show these features [21]. The presence of amyloid material favors the possibility of former. Immunohistochemistry and ultra structural examination are essential tools in diagnosing MTC [11] and differentiating it from melanoma in difficult situations. Calcitonin is indispensable in these situations. Immunohistochemical expression of S-100 protein and especially HMB-45 by the pigmented tumor cells is further proof of melanocytic differentiation. All the previously reported cases showed calcitonin and HMB-45 expression in tumor cells. Ikeda et al did not find HMB-45 positivity in the sustentacular-like pigmented cells, possibly due to loss of antigenicity secondary to prebleaching. These cells were however, positive for S-100 stain. Marcus et al demonstrated presence of melanin and neurosecretory granules by Fontana-Masson and Gremalius argyrophil silver stain. Most of the cases showed chromogranin positivity to support for neuroendocrine differentiation. Electron microscopy showing the premelanosomes and neurosecretory granules within the same tumor cells clinches the diagnosis of MTC with melanin production [3,4]. Melanoma does not contain neurosecretory granules and intermediate filaments of the cytokeratin type [22].

Melanin producing medullary carcinoma thyroid was first described by Marcus et al in 1982 [3] and since then eight cases have been reported in the literature [3-11] including one case from our institute [10]. Salient features are summarized in Table 1. Age ranged from 20 to 72 years with equal male to female ratio. Duration of symptoms varied from 15 days to 14 years. None of them showed c-cell hyperplasia. Interestingly all the reported cases showed variable histomorphological features. In the case reported by Marcus et al there was evidence of only scanty melanin production which was localized to dendritic cells which did not have neurosecretory granules [3] Similarly the case reported by Ikeda et al [9] showed paraganglioma like picture and pigment was localized to the sustentacular cells. The possibility of paraganglioma was not considered in view of calcitonin expression in these cells. In later studies it has been shown that melanosomes and neurosecretory granules are present in the same tumor cells [5]. The case reported by Kimura et al [6] showed glandular differentiation and both types of cells were pigmented.

We tend to agree that tumor arises by polyclonal evolution of a common neoplastic precursor cell, able to produce both melanin and calcitonin. These cells are derived from neural crest which has melanocytic and c-cell characteristics within the same cells or show a mixed phenotypic expression. This is further supported that some of pigmented medullary carcinomas showed paraganglioma like morphology [9] which were derivatives of neural crest.

The prognosis of this variant is not exactly known, due to scarcity of cases. Of the nine previously published cases, follow up was mentioned in five patients only. Of these, two patients showed systemic metastases in the follow up and one had lymph node metastasis at the time of presentation. The case under discussion had very high mitotic count in some foci and had recurrence thrice in the soft tissue of neck and metastases to skull bones within 24 months of surgery. The high mitotic rate as seen in this case may be associated with poor prognosis. Whether the presence of melanin contribute to its aggressiveness remains to be elucidated. Therefore more cases of such type need to be documented in order to predict the exact behavior of this variant.

## Conclusion

Melanocytic differentiation in medullary carcinoma of thyroid is very rare. The prognostic and therapeutic significance of this variant of MTC is not exactly known but this variant appears to be more aggressive; hence it needs to be documented in literature to know the exact behavior of this variant.

## Competing interests

The author(s) declare that they have no competing interests.

## Authors' contributions

KS, MCS and DJ are primarily responsible for drafting, literature search, and submission of the manuscript. RK supervised treatment. All authors have read and approved the final manuscript.
